# Effect of early rescue ICSI and split IVF‐ICSI in preventing low fertilization rate during the first ART cycle: A real‐world retrospective cohort study

**DOI:** 10.1002/rmb2.12420

**Published:** 2021-10-27

**Authors:** Linlin Jiang, Yifan Qian, Xiaoli Chen, Xiaohui Ji, Songbang Ou, Ruiqi Li, Dongzi Yang, Yu Li

**Affiliations:** ^1^ Department of Obstetrics and Gynecology Sun Yat‐Sen Memorial Hospital Sun Yat‐Sen University Guangzhou China

**Keywords:** early rescue ICSI, first ART cycle, low fertilization, short gamete coincubation, split IVF‐ICSI

## Abstract

**Purpose:**

To determine the utility of short gamete coincubation in *in vitro* fertilization (IVF‐S) combined with early rescue intracytoplasmic sperm injection (R‐ICSI) and split IVF‐ICSI in preventing low fertilization based on a retrospective cohort study.

**Methods:**

Couples with a high risk of low IVF fertilization during the first ART cycle underwent IVF‐S with R‐ICSI or split IVF‐ICSI. Fertilization rate, embryo quality, and clinical outcomes were measured.

**Results:**

After propensity score matching, we included 188 couples in the IVF‐S with R‐ICSI group as Group 1 and 720 in the split IVF‐ICSI group as Group 2. Normal fertilization rates were similar; however, Group 1 had a higher multiple pronuclei rate (10.42% vs. 4.50%, *p* < 0.001) but a higher embryo utilization rate (59.84% vs. 53.60%, *p* < 0.001). The groups were similar in the rates of high‐quality embryos, embryo implantation, clinical pregnancy, and live birth. Low IVF fertilization rate was 4.79% and 9.03% in Group 1 and Group 2, respectively, with similar fertilization rate and embryo development.

**Conclusion:**

IVF‐S with early R‐ICSI and split IVF‐ICSI were effective strategies in preventing low fertilization rate. IVF‐S with early R‐ICSI could become the preferred approach because of its advantages—higher embryo utilization rate, fewer ICSI procedures, similar clinical pregnancy rate, and live birth rate.

## 1 INTRODUCTION

The incidence of total fertilization failure in conventional *in vitro* fertilization (IVF) is about 3.52–20.00%,^1^, ^2^, ^3^ often leading to cycle cancellation. However, currently available diagnostic strategies are limited. Late rescue intracytoplasmic sperm injection (ICSI) at 16–18 h after fertilization could achieve better fertilization and cleavage rates, but subsequent embryonic development and pregnancy rates are not ideal due to eggs aging.^4^ There are presently two methods to prevent unpredicted low or failed fertilization, short gamete coincubation in IVF (IVF‐S) combined with early rescue ICSI (R‐ICSI) and IVF‐ICSI split insemination.

Oocytes have been observed to be fertilized 2~6 h after exposure to spermatozoa,^5^, ^6^ and the second polar body is released in ~90% of fertilized oocytes by 6 h using time‐lapse video cinematography.^7^ Short gamete coincubation while observing for early signs of fertilization makes it possible to perform early R‐ICSI attempts before the time‐related deterioration in oocyte quality.^8^, ^9^, ^10^, ^11^ However, to facilitate this observation, the granulosa cells around the oocyte should be removed under the microscope. Studies showed that IVF‐S had diverse effects on fertilization and embryo quality. Some studies showed that IVF‐S could reduce the adverse effects of the semen and its metabolites on the embryos, improving embryo quality and the clinical pregnancy rate.^12^, ^13^ Also, it has been reported that granulosa cell removal after six hours of short gamete coincubation had a lower abnormal fertilization rate than if they were removed at 20 h of fertilization.^14^ However, some studies reported that early removal of granulosa cells increased the multiple‐fertilization rate.^15^, ^16^, ^17^ Even so, most researchers believe that granulosa cell removal at six hours of short gamete coincubation enables early discovery of low or failed fertilization with no effect on embryo quality.^14^, ^17^, ^18^

IVF‐ICSI split insemination is another method to prevent fertilization failure.^19^, ^20^ Failed fertilization after IVF occurred in ten of 60 couples (16.7%) with unexplained infertility, but in none, if ICSI was performed with conventional IVF on sibling cumulus‐oocyte complexes (COCs).^19^ A committee opinion indicated that ICSI for unexplained infertility, without male factor infertility, was associated with a higher fertilization rate; however, it did not improve live birth outcomes.^21^ Some studies reported that IVF‐ICSI split insemination increased the fertilization rate and decreased the rates of complete fertilization failure in cases of mild male factor infertility^22^ and polycystic ovary syndrome (PCOS) patients.^23^ Although fertilization rate is higher through split IVF‐ICSI, the blastocyst formation rate obtained following ICSI was lower than after IVF.^24^

In the absence of clinical guidelines on the insemination method in couples with a high risk of fertilization failure in conventional IVF, methods were needed to prevent low fertilization rate. Till now, there were no studies that compared the effectivity of IVF‐S combined with early R‐ICSI and split IVF‐ICSI. In this study, we aimed to assess the effectivity of the two methods, trying to succeed in at least some cases of unpredictable fertilization failure, by real‐world retrospective cohort study.^25^

## 2 MATERIALS AND METHODS

### 2.1 Participants

A single‐center retrospective cohort study was performed, analyzing data of patients who underwent IVF‐S with early R‐ICSI (Group 1) or split IVF‐ICSI (Group 2) at the Sun Yat‐sen Memorial Hospital from January 2017 to July 2019. All patients were during their first assisted reproductive technologies (ART) cycle with four or more retrieved oocytes and at least one of the high‐risk factors for poor fertilization: infertility for more than five years, borderline semen parameters, or unexplained infertility. The diagnosis of unexplained infertility was based on the following criteria: regular ovulatory cycles, normal uterine cavity and patent fallopian tubes, no clinical or sonographic evidence of endometriosis, and normal semen parameters following the World Health Organization criteria. Patients with oocyte maturation disorder were excluded. The Sun Yat‐sen Memorial Hospital ethical review board approved the study. The data were anonymized, and the requirement for informed consent was therefore waived.

### 2.2 Clinical procedures

All participants were treated with a gonadotropin‐releasing hormone (GnRH) agonist or GnRH antagonist protocol. Individually adapted doses of the recombinant or highly purified follicle‐stimulating hormone were administered according to age and ovarian reserve, followed by adjusted dosage based on serum estradiol levels and follicular development, as indicated by ultrasonographic monitoring. Human chorionic gonadotropin (hCG) or GnRH agonist were administered to induce oocyte maturation when at least three follicles reached a mean diameter of 16 mm, or at least two follicles were 18 mm in diameter or larger. Oocytes were retrieved 36 h after triggering.

Semen was collected into sterile containers by masturbation and then kept for 30 minutes at 37°C. After liquefaction, samples were analyzed for sperm concentration and motility following the World Health Organization criteria. After liquefaction, sperms were selected by gradient centrifugation (Vitrolife, Sweden AB) and centrifuged at 500×*g* for 15 minutes. The sperm pellet was collected and washed in SpermRinse (Vitrolife, Sweden AB) at 300×*g* for 5 min and then IVF medium at 300×*g* for 5 min. The final sperm pellet was resuspended in 1 ml of IVF medium for insemination.

Before co‐incubation of the gametes, the rate of the progressive sperm cells was evaluated, and the oocytes were inseminated with 1.0 × 10^5^/ml progressive sperm cells 3–5 h after oocyte retrieval. In Group 1, four to five oocytes/zygotes, considered mature oocytes, with relatively loose cumulus cell layers, were selected by an experienced embryologist and transferred into GMOPS medium (Vitrolife, Sweden AB) for removing the cumulus cells 5 h post‐insemination. Then, the oocytes were transferred into G‐IVF medium (Vitrolife, Sweden AB) for polar body observation by inverted microscope (Nikon). The presence of two polar bodies indicated barrier‐free fertilization. If two or more oocytes were fertilized, the remaining oocytes/zygotes were cultured as in conventional IVF. If less than two metaphase II (MII) oocytes presented two polar bodies, the second evaluation for two polar body was done one more hour later for all oocytes. This study defined fertilization rate <30%, including total fertilization failure, as a low fertilization rate.^26^, ^27^ When a low fertilization rate was identified, early R‐ICSI was performed for unfertilized MII oocytes, which procedure was as same as regular ICSI, using the rest of the semen. COCs were randomly selected for ICSI or IVF in Group 2. The COCs were preincubated for 3–6 h before insemination or injection. Conventional IVF and ICSI were performed according to the laboratory's routine insemination procedures. The oocytes in the standard incubation time group were co‐incubated with sperm in 1 mL of IVF medium for 16–18 h.

Embryo quality was classified based on the embryo morphology following the Istanbul consensus workshop on embryo assessment.^28^ D3 cleavage embryos or blastocysts were transferred. All patients received the luteal support with progesterone from the day of oocyte retrieval. On Day 14 ± 3 after ET, participants underwent a pregnancy test (serum measurement of β‐hCG). Clinical pregnancy was confirmed through ultrasonographic observation of the intrauterine gestation sac, the fetal pole, and cardiac activity at 6–7 weeks of gestation. If pregnancy was confirmed, luteal phase support would have continued for up to ten weeks of gestation. The neonatal outcome data were obtained by telephone interview with the parents after delivery.

### 2.3 Statistical analysis

#### 2.3.1 Case matching

The groups differed in age and sperm concentration after recruiting based on the inclusion and exclusion criteria. We included the female age and sperm concentration in 1:4 propensity score matching (PSM) to eliminate the influence of baseline differences in this real‐world study, as these might act as confounding factors.

#### 2.3.2 Statistical method

R 3.5.3 software (The University of Auckland, Auckland city, New Zealand) was used for PSM. IBM SPSS Statistics for Windows, Version 24.0 software (IBM Corp.) was used for data analysis. PASS (Power Analysis and Sample Size) 15.0.5 was used for power analysis based on sample size and live birth rate of two groups. Continuous variables are expressed as mean ± standard deviation, and the independent sample *t*‐test compared the groups. Count data are expressed as percentage (%), and the groups were compared for rates by chi‐square test or Fisher's exact test. Differences with *p* < 0.05 were considered statistically significant.

## 3 RESULTS

We identified 191 cases of Group 1 and 775 cases of Group 2 based on the inclusion and exclusion criteria. After PSM, Group 1 comprised 188 cases and group 2 consisted of 720 cases, as shown in Table 1. The data in Table 1 show that Group 2 had a longer infertility period, but the proportion of primary infertility was lower than in Group 1. The proportion of GnRH agonist protocol was higher in Group 1 compared with Group 2. The mean patient age, etiology of infertility, baseline serum FSH, AMH and sperm concentration, and motility on oocyte retrieval day were similar between the groups after PSM.

**TABLE 1:** Demographic characteristics of the patients in the two study groups

	Before PSM		*p*‐value	After PSM		*p*‐value
	Group 1	Group 2		Group1	Group 2	
	*N* = 191	*N* = 775		*N* = 188	*N* = 720	
Age (y)	31.37 ± 3.85	32.34 ± 3.96	**0.002**	31.46 ± 3.79	31.94 ± 3.72	0.112
Years of infertility (y)	4.89 ± 2.66	5.96 ± 3.20	**0.000**	4.93 ± 2.66	5.89 ± 3.15	**0.000**
Primary infertility (%) Infertility factors, % (*n*)	78.53 (150/191)	67.48 (523/775)	**0.003**	78.72 (148/188)	69.58 (501/720)	**0.013**
Ovulation dysfunction	9.42(18/191)	10.71 (83/775)	0.131	9.57 (18/188)	10.98 (79/720)	0.089
Tubal factor	21.99 (42/191)	26.58 (206/775)		21.28 (40/188)	27.5 (198/720)	
Endometriosis	4.71 (9/191)	3.23 (25/775)		4.79 (9/188)	3.06 (22/720)	
Mild male factor	16.75 (32/191)	15.23 (118/775)		16.49 (31/188)	14.58 (105/720)	
Unexplained factor	27.74 (53/191)	22.19 (172/775)		28.19 (53/188)	22.50 (162/720)	
Multiple female factor	10.99 (21/191)	8.13 (63/775)		11.17 (21/188)	7.92(57/720)	
Both female and male	8.38 (16/191)	13.94 (108/775)		8.51 (16/188)	13.47 (97/720)	
Basal FSH (U/L)	8.05 ± 3.33	7.88 ± 3.91	0.164	8.06 ± 3.35	7.83 ± 3.90	0.458
AMH (ng/ml)	5.03 ± 3.85	5.40 ± 3.83	0.372	4.98 ± 3.79	5.54 ± 3.87	0.151
COS protocol, % (*n*)						
GnRH agonist	73.82 (141/191)	65.16 (505/775)	**0.023**	73.94 (139/188)	64.86 (467/720)	**0.019**
GnRH antagonist	26.18 (50/191)	34.84 (270/775)		26.06 (49/188)	35.14 (253/720)	
Duration of stimulation	11.38 ± 2.47	11.01 ± 2.87	0.102	11.40 ± 2.48	11.01 ± 2.93	0.097
Gn (IU)	2109 ± 865	2003 ± 774	0.097	2122 ± 864	1982 ± 768	**0.03**
Semen concentration(M/ml)	63.07 ± 36.39	56.63 ± 35.04	**0.02**	61.73 ± 33.97	57.74 ± 33.34	0.145
Semen motility (%)	55.47 ± 12.15	53.87 ± 12.80	0.118	55.61 ± 11.74	53.88 ± 12.84	0.093
Sperm total motile count (M)	94.48 ± 74.07	85.75 ± 81.40	0.177	93.78 ± 73.35	87.44 ± 81.23	0.332

Note

Data are expressed as mean ± standard deviation or percentage. Bold fonts highlight statistical significance (*p* < 0.05). Group 1, short gamete coincubation during *in vitro* fertilization (IVF) with early rescue intracytoplasmic sperm injection (ICSI); Group 2, split IVF and ICSI.

Abbreviations: AMH, Anti‐miüllerian hormone; basal FSH, basal follicle‐stimulating hormoe; COS, controlled ovarian hyperstimulation; Gn, gonadotropin; M, million; PSM, propensity score matching.

In Group 1, five cases were under total IVF fertilization failure and nine cases were under low IVF fertilization which were performed early R‐ICSI. In Group 2, 32 cases were under total IVF fertilization failure and 65 cases were under low IVF fertilization. As shown in Table 2, similar complete IVF fertilization failure (2.66% vs. 4.44%) and low IVF fertilization (4.79% vs. 9.03%) rates were noted in Groups 1 and 2, respectively. The groups were also similar in the rates of normal fertilization following IVF, normal cleavage following IVF, overall normal fertilization, overall normal cleavage, d3 high‐quality embryos, blastocyst formation, high‐quality blastocyst, embryo implantation, clinical pregnancy, miscarriage, loss to follow‐up, and birth. However, rates of multiple pronuclei (PN) following IVF and overall multiple PN in Group 1 were significantly higher than in Group 2. The rate of embryo utilization was 59.84% in Group 1 and 53.60% in Group 2 (*p* < 0.001).

**TABLE 2:** Fertilization rate, embryo quality, and clinical outcomes in the study groups

	Group 1 *n* = 188	Group 2 *n* = 720	*χ* ^2^ value	*p*‐value
Total IVF fertilization failure, % (*n*)	2.66 (5/188)	4.44 (32/720)	1.215	0.270
Low IVF fertilization, % (*n*)	4.79 (9/188)	9.03 (65/720)	3.581	0.058
*n* (oocytes)	2333	10035		
IVF fertilization rate (2PN), % (*n*)	57.31 (1337/2333)	58.00 (2814/4852)	0.306	0.580
IVF >2PN rate, % (*n*)	10.42 (243/2333)	8.41 (408/4852)	7.701	**0.006**
IVF normal cleavage rate, % (*n*)	97.16 (1299/1337)	97.58 (2746/2814)	0.660	0.417
Fertilization rate (2PN), % (*n*)	58.81 (1372/2333)	59.12 (5933/10035)	0.078	0.781
>2PN rate, % (*n*)	10.42 (243/2333)	4.50 (452/10035)	124.728	**0.000**
Normal cleavage rate, % (*n*)	97.23 (1334/1372)	97.30 (5773/5933)	0.022	0.881
D3 high‐quality cleavage embryo rate, % (*n*)	32.08 (428/1334)	30.63 (1768/5773)	1.080	0.299
Blastocyst formation rate, % (*n*)	60.03 (479/798)	59.33 (1886/3179)	0.129	0.719
High‐quality blastocyst rate, % (*n*)	18.79 (90/479)	16.01 (302/1886)	2.129	0.144
*n* (embryo transferred cycles)	142	503		
*n* (embryo transferred)	276	907		
Stage of embryos transferred, % (n)				
D3 cleavage embryos	95.77 (136/142)	90.85 (457/503)	3.616	0.057
Blastocyst	4.23 (6/142)	9.15 (46/503)		
Implantation rate, % (*n*)	41.30 (114/276)	38.15 (346/907)	0.887	0.346
Clinical pregnancy rate, % (*n*)	56.33 (80/142)	53.28 (268/503)	0.417	0.519
Miscarriage rate, % (*n*)	10.00 (8/80)	11.94 (32/268)	0.228	0.633
Early miscarriage rate, % (*n*)	3.75 (3/80)	8.21 (22/268)	1.837	0.175
Loss to follow‐up rate, % (*n*)	2.50 (2/80)	1.49 (4/268)	0.369	0.544
Live birth rate, % (*n*)	49.30 (70/142)	43.94 (221/503)	1.285	0.257

Note

Data are expressed as percentage. Bold fonts highlight statistical significance (*p* < 0.05). Group 1, short gamete coincubation during *in vitro* fertilization (IVF) with early rescue intracytoplasmic sperm injection (ICSI); Group 2, split IVF and ICSI. PN, pronuclei.

We divided the study patients into low (*n* = 74) and normal (*n* = 834) fertilization rate following IVF, using the 30% as a cut‐off value. Table 3 shows the baseline characteristics of these two groups. The groups were similar for age, years of infertility, primary infertility rate, etiology of infertility, basal FSH, AMH, COS protocol, sperm concentration, and sperm motility.

**TABLE 3:** The baseline characteristics of low and normal IVF fertilization rate groups

	Low IVF fertilization rate	Normal IVF fertilization rate	*p*‐value
*n*	74	834	
Age (y)	31.86 ± 3.37	31.84 ± 3.77	0.959
Years of infertility (y)	5.49 ± 2.87	5.71 ± 3.09	0.567
Primary infertility (%) Infertility factors, % (*n*)	77.03 (57/74)	70.98 (592/834)	0.270
Ovulation dysfunction	6.76 (5/74)	11.03 (92/834)	0.634
Tubal factor	25.68 (19/74)	26.26 (219/834)	
Endometriosis	4.05 (3/74)	3.36(28/834)	
Mild male factor	14.86 (11/74)	14.99 (125/834)	
Unexplained factor	22.97 (17/74)	23.74 (198/834)	
Multiple female factor	6.76 (5/74)	8.75 (73/834)	
Both female and male	18.92 (14/74)	11.87 (99/834)	
Basal FSH (U/L)	7.47 ± 2.34	7.92 ± 3.89	0.332
AMH (ng/ml)	6.33 ± 4.32	5.33 ± 3.80	0.079
COS protocol, % (*n*)			
GnRH agonist	59.46 (44/74)	67.39 (562/834)	0.165
GnRH antagonist	40.54 (30/74)	32.61 (272/834)	
Duration of stimulation	10.88 ± 2.56	11.11 ± 2.87	0.499
Gn (IU)	1956 ± 811	2016 ± 789	0.53
Sperm concentration (M/ml)	53.61 ± 37.80	59.00 ± 33.08	0.238
Sperm motility (%)	52.50 ± 12.96	54.39 ± 12.60	0.218
Sperm total motile count (M)	72.07 ± 61.91	90.24 ± 80.92	0.060

Note

Data are expressed as mean ± standard deviation or percentage.

Abbreviations: basal FSH, basal follicle‐stimulating hormoe; IVF, *in vitro* fertilization; M, million.

Low fertilization following IVF occurred in 9 cases in Group 1 and 65 cases in Group 2. In Group 1, only 7 oocytes were fertilized among 92 oocytes retrieved undergoing IVF‐S. Then, 47 unfertilized MII oocytes were done R‐ICSI. In Group 2, 51 oocytes were fertilized among 417 oocytes by IVF and 231 oocytes were fertilized among 331 MII oocytes by ICSI. These two subgroups were similar for basic characteristics, including age, years of infertility, primary infertility rate, causes of infertility, and semen motility, as shown in Table 4. However, sperm concentration in couples from Group 1 was slightly lower than in the couples from Group 2. The subgroups were similar for rates of fertilization following IVF, ICSI normal fertilization, cleavage, d3 high‐quality embryos, blastocyst formation, high‐quality blastocyst, and embryo utilization. Fourteen embryos were transferred into seven transfer cycles of the couples from Group 1, and 74 embryos were transferred into 44 transfer cycles of the couples from Group 2. The data in Table 4 show that rates of embryo transfer cancellation, implantation, clinical pregnancy, and live birth were similar in the two subgroups.

**TABLE 4:** Baseline characteristics, fertilization rate, embryo development, and clinical outcomes of early R‐ICSI in Group 1 and ICSI in Group 2 for couples with low IVF fertilization rate

	Early R‐ICSI in Group 1	ICSI in Group 2	*p*‐value
*n*	9	65	
Age (y)	32.67 ± 4.39	31.75 ± 3.24	0.451
Years of infertility (y)	5.22 ± 3.46	5.53 ± 2.81	0.765
Primary infertility (%) Infertility factors %(n)	77.78 (7/9)	76.92 (50/65)	0.954
Ovulation dysfunction	11.11 (1/9)	6.15(4/65)	1.000
Tubal factor	33.33 (3/9)	24.62(16/65)	
Endometriosis	0(0/9)	4.62 (3/65)	
Mild male factor	11.11 (1/9)	15.38 (10/65)	
Unexplained factor	22.22 (2/9)	23.08 (15/65)	
Multiple female factor	0(0/9)	7.69 (5/65)	
Both female and male	22.22 (2/9)	18.46 (12/65)	
*n* (oocytes)	92	865	
*n* (ICSI MII oocytes)	47	331	
Sperm concentration (M/ml)	30.56 ± 21.42	56.80 ± 38.57	**0.007**
Sperm motility (%)	47.22 ± 14.39	53.23 ± 12.70	0.194
Sperm total motile count (M)	37.67 ± 27.67	76.84 ± 63.92	**0.004**
IVF fertilization rate, % (*n*)	7.61(7/92)	12.23 (51/417)	0.207
ICSI fertilization rate (2PN), % (*n*)	74.47 (35/47)	69.79 (231/331)	0.511
ICSI normal cleavage rate, % (*n*)	100.00 (35/35)	97.40 (225/231)	0.335
ICSI fertilization rate (>2PN), % (*n*)	2.13 (1/47)	0.91 (3/331)	0.444
D3 high‐quality cleavage embryo rate, % (*n*)	38.46 (15/39)	33.59 (88/262)	0.549
Blastocyst formation rate, % (*n*)	20.00 (2/10)	54.37 (56/103)	0.081
High‐quality blastocyst rate, % (*n*)	0.00 (0/2)	16.07 (9/56)	1.000
*n* (embryo transferred cycle)	7	44	
*n* (embryo transferred)	14	74	
Stage of embryos transferred, % (*n*)			
D3 cleavage embryos	100.00 (7/7)	90.91 (40/44)	1.000
Blastocyst	0.00 (0/7)	9.09 (4/44)	
Embryo transfer cancellation rate, % (*n*)	22.22 (2/9)	32.31 (21/65)	0.540
Implantation rate, % (*n*)	57.14 (8/14)	41.89 (31/74)	0.292
Clinical pregnancy rate, % (*n*)	71.43 (5/7)	52.27 (23/44)	0.344
Live birth rate, % (*n*)	71.43 (5/7)	40.91 (18/44)	0.132

Note

Data are expressed as mean ± standard deviation or percentage. Bold fonts highlight statistical significance (*p* < 0.05). Group 1, short gamete coincubation during *in vitro* fertilization (IVF) with early R‐ICSI; Group 2, split IVF and ICSI.

Abbreviations: ICSI, intracytoplasmic sperm injection; M, million; MII, metaphase II; PN, pronuclei; R‐ICSI, rescue ICSI.

## 4 DISCUSSION

After PSM, this study included 188 couples in IVF‐S with early R‐ICSI and 720 in split IVF‐ICSI. Total fertilization failure occurred in 37 of 908 couples (4.07%), and a low IVF fertilization rate was noted in 74 of 908 couples (8.15%). Early R‐ICSI benefited 4.79% of patients in Group 1, and ICSI benefited 9.03% of patients in Group 2. Even though Group1 had a higher multiple pronuclei rate, the groups were similar in rates of d3 high‐quality embryos, blastocyst formation, high‐quality blastocyst, embryo implantation, clinical pregnancy, miscarriage, loss to follow‐up, and birth. What is more, Group 1 had a higher embryo utilization rate. Nine cases were done R‐ICSI in Group 1, and 65 cases were performed split IVF‐ICSI in Group2 based on low IVF fertilization rate. Similar fertilization rate, embryo development, and clinical outcomes were noted between these two groups.

Total fertilization failure or low fertilization rate after conventional IVF is still prevalent and unpredictable. It is not surprising that we found no difference between the low and normal IVF fertilization rate groups, even though we believe that infertility for over five years, borderline sperm quality, or unexplained infertility are high‐risk factors for poor fertilization. Early R‐ICSI prevented complete fertilization failure. The rates of fertilization, implantation, and pregnancy in early R‐ICSI after IVF‐S were much higher than the so‐called ICSI rescue procedure in which ICSI is performed on the second day.^10^ Split IVF‐ICSI randomly allocates the obtained oocytes to IVF or ICSI, preventing in part the risk of complete fertilization failure or low fertilization rate. Here, we showed higher fertilization rate for early R‐ICSI compared with IVF‐S in Group 1 with low fertilization rate. Also, in Group 2 with low fertilization rate, split ICSI displayed higher fertilization rate compared with split IVF. Therefore, we demonstrated that early R‐ICSI and split IVF‐ICSI were effective strategies in preventing IVF fertilization failure or low fertilization rate in couples with high‐risk factors.

Our results documented that normal fertilization rates in the two groups were similar; however, a higher multiple PN rate was noted in Group 1 (10.42% vs. 8.41%). The effect of early granulosa cell removal on multiple PN incidence remains controversial. Ding et al. found that the normal fertilization rate when removing the granulosa cells after incubating the gametes for five hours was similar to conventional IVF, but the proportion of three PN embryos was significantly higher (10.9% vs. 4.5%).^29^ Other studies had similar findings.^15^, ^17^ However, He et al. found no difference in multiple PN rate,^30^ and Xiong et al. reported lower multiple PN rate (7.48% vs. 9.22%)^14^ in IVF‐S than conventional IVF. Because granulosa cells provide the oocyte with growth factors or adhesion molecules and play an important role in oocyte growth, maturation, and normal fertilization, early removal of granulosa cells might affect the second meiosis or cortical reaction during IVF. Another explanation for higher multiple PN rate might be that a tiny minority of fertilized oocytes recognized as unfertilized, were performed R‐ICSI. One of the most critical parts of the present study was to identify within six hours after conventional insemination those eggs that fail to fertilize. Therefore, one must rely on the correct identification of polar bodies. However, the eggs may be still under the process of fertilization despite the absence of 2PB after six hours of IVF incubation, which could lead to abnormal fertilization after R‐ICSI. Payne et al showed that the second polar body was released in ~90% of fertilized oocytes by 6 h and the maximum time course for 2PB extrusion was 8 h which had very small percentage by time‐lapse incubator.^7^ Meanwhile, another study, which did 2PB evaluation after 6 h incubation, showed that oocytes which were identified with one polar body but were not reinseminated by ICSI (50 eggs) did not develop pronuclei and did not show any other signs of fertilization.^9^ Therefore, using six hours for observation of 2PB is convinced, even though a tiny minority of fertilized oocytes might be recognized as unfertilized. In such situation, time‐lapse incubators will be performed in the future study for identifying fertilization. One interesting finding in this study was that the multiple PN rate following IVF‐S in Group 1 was 10.42%, slightly lower than the 12.6 and 13.34% reported in other studies.^15^, ^17^ A possible explanation for this difference could be the fact that the early granulosa cell removal after short gamete coincubation in our study was performed in only four or five oocytes/zygotes per patient, rather than all oocytes/zygotes. If two or more oocyte/zygotes were fertilized, the rest were cultured as conventional IVF. This reduced the proportion of early granulosa cells removal.

What is more, this study showed that the effectiveness of early R‐ICSI after IVF‐S was similar to conventional ICSI. As shown in Table S1, early R‐ICSI and the conventional ICSI in Group 2 were similar in the rates of normal fertilization, cleavage, d3 high‐quality cleavage embryo, and high‐quality blastocyst. It was important to show that clinical pregnancy rate, implantation rate, and live birth rate were comparable between early R‐ICSI and regular ICSI.^10^ Meanwhile, early R‐ICSI was shown to be safe for the offspring. A study with large number cases was to investigate the effects of early cumulus cell removal (n = 570) on pregnancy and neonatal outcomes as compared to routine cumulus cell removal (n = 1214), and it showed that early cumulus cell removal alone was not associated with adverse pregnancy and neonatal outcomes.^31^ Also, no differences in sex, birth weight, and birth defects were found between newborns delivered by conventional IVF, ICSI, or early R‐ICSI.^32^, ^33^ However, possible risks associated with the extra manipulation during fertilization assessment after IVF‐S still exist. Therefore, only four or five oocytes/zygotes per patient, rather than all oocytes/zygotes, were chosen for polar body observation. The method to reduce proportion of early granulosa cells removal might help attenuate the risks.

Here, we suggest that early R‐ICSI and split IVF‐ICSI are effective methods to reduce IVF fertilization failure or low fertilization in couples with high‐risk factors for poor fertilization. We believe that split IVF‐ICSI is effective for high‐risk patients in the first ART, but in this method, more oocytes are fertilized by ICSI. From the economic^34^ and offspring safety perspectives,^35^, ^36^ early R‐ICSI is preferred. Although this approach increased the multiple PN incidence, it was similar to split IVF‐ICSI in rates of normal fertilization, normal cleavage, high‐quality embryo, clinical pregnancy, and birth. The limitation of this study is a retrospective design. Here, we use propensity score matching to eliminate the influence of baseline differences in this real‐world study. Another limitation of this study is the relatively low statistical power (25.87%), which may be due to the small sample size. Larger sample size from multiple centers is needed. Also, it would be better to do randomized controlled studies and include IVF group as control group.

In conclusion, IVF‐S with early R‐ICSI and split IVF‐ICSI are both effective strategies in preventing IVF fertilization failure or low fertilization rate in couples with high‐risk factors. IVF‐S with early R‐ICSI could become the preferred approach because of its advantages—higher embryo utilization rate, fewer ICSI procedures, similar clinical pregnancy rate, and similar live birth rate.

## CONFLICT OF INTEREST

All authors declared no conflict of interest.

## HUMAN RIGHTS STATEMENTS AND INFORMED CONSENT

The data were anonymized, and the requirement for informed consent was therefore waived.

## ETHICAL APPROVAL

All procedures performed in this study followed the ethical standards of the Ethical Review Board (ERB) of Sun Yat‐sen Memorial Hospital (No. SYSEC‐KY‐KS‐2021–123).

## Supporting information

Table S1
