# Supplementary material for: Effect of early rescue ICSI and split IVF‐ICSI in preventing low fertilization rate during the first ART cycle: A real‐world retrospective cohort study
Source: Reprod Med Biol. 2021 Oct 27;21(1):e12420. doi: 10.1002/rmb2.12420 (PMC8656193; doi:10.1002/rmb2.12420)
Supplement: Supplementary file 1 — Table S1 [file RMB2-21-e12420-s001.docx]

Table S1 Comparison of fertilization and embryo development following early R-ICSI in Group 1 and ICSI in Group 2

|  | Early R-ICSI in Group 1 | ICSI in Group 2 | *P*-value |
| --- | --- | --- | --- |
| *n* | 9 | 720 |  |
| Age (y) | 32.67±4.39 | 31.94±3.72 | 0.564 |
| Years of infertility (y) | 5.22±3.46 | 5.89±3.14 | 0.527 |
| *n* (MII oocyte) | 47 | 4281 |  |
| Sperm concentration (M/mL) | 30.56±21.42 | 57.74±33.3 | **0.015** |
| Sperm motility (%) | 47.22±14.39 | 53.88±12.84 | 0.123 |
| Sperm total motile count (M) | 37.67±27.67 | 87.44±81.23 | 0.067 |
| IVF fertilization rate, % (*n*) | 7.61(7/92) | 72.88(3536/4852) | **0.000** |
| ICSI fertilization rate (2PN), % (*n*) | 74.47(35/47) | 73.18(3119/4281) | 0.805 |
| ICSI fertilization rate (>2PN), % (*n*) | 2.13(1/47) | 1.03(44/4281) | 0.460 |
| ICSI normal cleavage rate, % (*n*) | 100(35/35) | 97.05(3027/3119) | 0.302 |
| D3 high-quality cleavage embryo rate, % (*n*) | 38.46(15/39) | 30.63(1768/5773) | 0.290 |
| Blastocyst formation rate, % (*n*) | 20.00(2/10) | 59.33(1886/3179) | **0.028** |
| High-quality blastocyst rate, % (*n*) | 0.00(0/2) | 16.02(302/1886) | 1.000 |

Data are expressed as mean ± standard deviation or percentage. Bold fonts highlight statistical significance (*P* < 0.05).

ICSI, intracytoplasmic sperm injection; R-ICSI, rescue ICSI; M, million; MII, metaphase II; PN, pronuclei. Group 1, short gamete coincubation during *in vitro* fertilization (IVF) with early R-ICSI; Group 2, split IVF and ICSI.
